# Watt-level high-OSNR continuous wave tunable figure-8 holmium-doped fiber laser for OWC systems

**DOI:** 10.1371/journal.pone.0334590

**Published:** 2025-10-30

**Authors:** Ammar Armghan, Ahmad Atieh, Khaled Aliqab, Meshari Alsharari, Jawad Mirza, Slim Chaoui, Muhammad Ijaz

**Affiliations:** 1 Department of Electrical Engineering, College of Engineering, Jouf University, Sakaka, Saudi Arabia; 2 Optiwave Systems Inc., Ottawa, Ontario, Canada; 3 Department of Electrical Engineering, HITEC University Taxila, Pakistan; 4 SEECS Photonics Research Group, Islamabad, Pakistan; 5 Department of Computer Engineering and Networks, College of Computer and Information Sciences, Jouf University, Sakaka, Saudi Arabia; 6 Department of Engineering, Manchester Metropolitan University, Manchester, United Kingdom; Zhejiang University, CHINA

## Abstract

The 2000 nm optical transmission window is gaining significant interest for terrestrial and deep-space optical wireless communication (OWC) due to its exceptionally low atmospheric absorption. Therefore, high-power Holmium-doped fiber lasers (HDFLs) with high optical signal-to-noise ratio (OSNR) are attractive solutions for OWC systems because they can enhance transmission efficiency, improve signal quality over longer transmission distances, and ensure reliable communication in challenging atmospheric conditions by operating in the eye-safe 2000 nm wavelength range. In this work, we propose the design of a watt-level, high-OSNR continuous wave (CW) HDFL tunable in 2022-2140 nm wavelength range based on figure-8 (F8) cavity and a single in-band backward pump source. The performance of F8 HDFL is evaluated considering optimized pumping configuration, length of Holmium-doped fiber (HDF), and Ho^3+^ ions density. Maximum output powers of 0.842 W, 1.5 W, and 2 W are obtained at lasing wavelength of 2046.7 nm for 20%, 40%, and 60% of coupling ratios, respectively. Highest slope efficiency (SE) of 51.8% is achieved at 2046.7 nm for 60% of coupling ratio considering optimized parameters. OSNR and linewidth (LW) in the range of 30.4-93.3 dB and 18.5-19.9 MHz, respectively are obtained when laser is tuned in 2022-2140 nm wavelength range. A power fluctuation around 0.3 dB at lasing wavelengths of 2022 nm and 2100 nm for fifteen iterations, each taken at five-minute interval is observed for 20% coupling ratio. Finally, the effect of pair induced quenching (PIQ) at laser’s output power is analyzed. A negligible penalty of around 7 mW is observed in output power of the laser at 2046.7 nm for 20% coupling ratio.

## 1 Introduction

Fiber lasers operating around 2000 nm eye-safe optical window have gained significant research interest due to their diverse applications in areas such as remote sensing, free-space optical communication, and medical procedures [1,2]. Therefore, watt-level fiber laser systems must be designed carefully for transportability, operator eye-safety, and the ability to concentrate their maximum power into a precise spot. Attaining these demanding features necessitates a fiber laser that is not only compact and robust against environmental effects like vibrations and temperature variations but also highly efficient with power conversion efficiency typically over 20% [3]. Additionally, essential performance criteria include emission at a wavelength greater than 1400 nm to ensure both atmospheric transmission and eye-safety, operation in a single spatial mode preferably Gaussian for superior beam quality, and minimal heat generation [3]. Finally, the capability for narrow linewidth amplification is indispensable, as it enables the coherent combination of multiple laser sources to achieve even greater power. A prominent advantage of high-power fiber lasers emitting in 2000–2200 nm wavelength range compared to conventional 1550 nm counterparts, lies in their suitability for deep-space optical links. This is primarily attributed to reduced Mie scattering and lower atmospheric absorption at longer wavelengths [1,4]. The gain media for such lasers are typically doped with Tm^3+^, Ho^3+^ ions, or a combination of both [1,4]. While Thulium-doped fibers (TDFs) exhibit peak emission around 1900 nm, their efficiency gradually decreases beyond 2000 nm due to the low emission cross-section of Tm^3+^ ions [4]. Efficient laser emission at wavelengths above 2000 nm can be effectively achieved using HDFs which are typically pumped by Thulium-doped fiber lasers (TDFLs) [5]. In the context of OWC systems, high OSNR and power HDFLs operating beyond 2000 nm are of critical importance. Their emission in the eye-safe spectral window combined with reduced atmospheric attenuation and scattering, makes them ideal for long-range, high-capacity free-space optical links. High output power ensures sufficient signal strength over extended distances, while a high OSNR is essential for maintaining signal integrity and minimizing the Bit-error rate (BER) in bandwidth-intensive communication scenarios. Therefore, HDFLs with optimized power and spectral purity represent a key enabling technology for next-generation high-performance OWC systems.

The F8 cavity design enhances the OSNR and enables high optical power generation through its unique nonlinear loop mirror configuration which acts as an effective mode-locking and noise-suppressing element [6,7]. This geometry supports unidirectional operation and self-pulsing behavior, reducing amplified spontaneous emission (ASE) noise and enhancing the spectral purity [6]. Additionally, the cavity’s bidirectional structure allows efficient recycling of intracavity power, leading to improved gain utilization and higher output power [6,8]. These characteristics make F8 cavities suitable for generating high-power, low-noise laser output required in demanding applications such as OWC systems.

### 1.1 Related work

Fiber lasers based upon F8 cavities have been widely proposed in past works. For instance, F8 Ytterbium-doped fiber lasers for generation of solitons [9], spatiotemporal mode-locked pulses [10], continuous wave lasers [11], and switchable and adjustable spacing multiwavelength nanosecond pulses [12], F8 Erbium-doped fiber lasers for studying the degree of polarization [13], generation of variable width pulses [14], generation of dissipative-soliton resonance [15], generation of stable noise-like pulses with varying dispersion [16], generation of widely tunable single and dual wavelengths [17], generation of mode-locked pulses [18], and generation of dual-comb [19], F8 Thulium-doped fiber laser for generation of noise-like pulses [20,21] and mode-locked pulses [22], and F8 Holmium-doped fiber laser for generation of noise-like pulses [23] and dissipative solitons [24]. Table 1 further elaborates the literature review by comparing the main accomplishments of past studies with the proposed work.

**Table 1:** Summary of literature review and comparison of main results with the proposed F8 HDFL.

Study	No. of pumps	Tuning wavelength	OSNR	Output power
[9]	2, Forward	1070 nm	-	-
[10]	1, Forward	1030-1080 nm	-	-
[11]	2, Backward	1030 nm	-	41.5 mW
[12]	1, Forward	1040-1063 nm	-	30 mW
[13]	1, Forward	1535-1545 nm	-	-
[15]	1, Backward	1565-1603 nm	-	20.2 mW
[16]	1, Forward	1560 nm	-	29.8 mW
[17]	1, Backward	1558-1605 nm	-	53.3 mW
[19]	1, Backward	1560 nm	-	-
[20]	1, Forward	1943 nm	64 dB	36.2 mW
[21]	2, Bidirectional	2034 nm	-	-
[22]	1, Forward	2034 nm	-	1000 mW
[23]	2, Bidirectional	2106.9 nm	-	5330 mW
[24]	2, Forward (dual-stage)	2103.7 nm	-	233 mW
Proposed	1, Backward	2022-2140 nm	93.3 dB	2000 mW

From the comprehensive literature survey presented above, it is evident that tunable HDFL based on F8 cavity operating in CW regime has not been proposed earlier. Therefore, we demonstrate for the very first time a watt-level, high-OSNR tunable HDFL operating in 2022-2140 nm wavelength range based on F8 cavity and single in-band backward pump source using numerical simulations. The laser generates up to 2 W output power at 2046.7 nm with a maximum SE of 51.8% considering optimized pumping configuration, fiber length, and Ho^3+^ concentration. The laser maintains an OSNR and LW in the range of 30.4–93.3 dB and 18.5–19.9 MHz across the entire tuning range, respectively.

Based on above details, the main achievements and significance of this research are as under:

- Demonstration of a watt-level, high-OSNR CW tunable HDFL operating in 2022-2140 nm wavelength range using numerical simulations.
- The laser is implemented using F8 cavity and single in-band backward pump source.
- Optimization process yields HDF length of 20 m, doping concentration of 60×10^24^ m^−3^, and backward pumping as optimized parameters.
- Maximum output powers of 2 W is obtained at lasing wavelength of 2046.7 nm for 60% of coupling ratios using a single in-band backward 5 W pump source.
- Highest SE of 51.8% is achieved at 2046.7 nm for 60% of coupling ratio considering optimized parameters.
- OSNR and linewidth in the range of 30.4-93.3 dB and 18.5-19.9 MHz, respectively are obtained corresponding to 2020-2140 nm wavelength range.
- Exceptional operational stability with minimal power fluctuation < 0.3 dB is observed.

This work is implemented using OptiSystem 22 software developed by Optiwave Inc [25]. The remainder of this manuscript is ordered as described below. Sect 2 provides a theoretical overview of the proposed design, Sect 3 discusses the optimization of the proposed laser, simulation setup is discussed in Sect 4, followed by the results in Sect 5. Finally, Sect 6 concludes the paper.

## 2 Background theory

To completely understand the operating mechanism of the proposed tunable F8 HDFL operating in CW regime, it is essential to understand the dynamics of Ho^3+^ ions doped in Silica host through spectroscopic analysis along with the configuration of the F8 cavity.

### 2.1 Spectroscopic analysis of Ho^3+^

**Fig 1:**
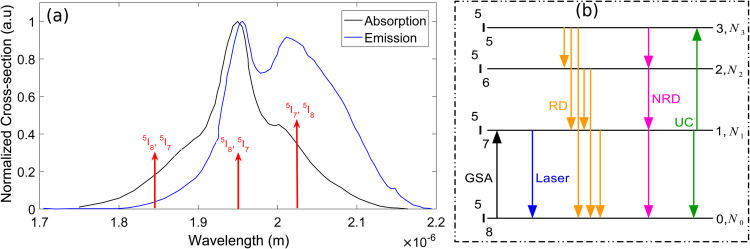
(a) Holmium’s absorption and emission cross-section (b) four level energy diagram [26].

Fig 1 shows the normalized absorption and emission cross-section spectra of Ho^3+^ ions in Silica host along with a four-level energy diagram illustrating the most commonly occurring transitions [26]. The Ho^3+^ ions exhibit a broad absorption band extending from 1800–2100 nm with maximum absorption at approximately 1950 nm [27,28]. In practical implementations, these ions are efficiently excited through in-band pumping at either 1950 nm or 1840 nm wavelengths, typically achieved using TDFL based pump sources. The Ho^3+^ ions residing at ground energy state ${\;\;}^{5}I_{8}$ are excited to ${\;\;}^{5}I_{7}$ level through ground state absorption (GSA) using in-band pumping as mentioned above. The transition governing the GSA and corresponding lasing at 2000 nm is ${\;\;}^{5}I_{7}\to^{5}I_{8}$. This GSA process and the corresponding lasing transition are clearly indicated by red arrows at the absorption and emission spectra of Fig 1a. The ion-ion interaction process in Holmium-doped systems is a nonradiative energy transfer mechanism between excited Ho^3+^ ions, commonly referred to as ion clustering [29]. This phenomenon is mainly governed by homogeneous upconversion (UC). It detrimentally impacts the performance of HDFLs, as it promotes one Ho^3+^ ion to a higher energy state while demoting another to a lower energy level resulting in reduced efficiency and increased losses [29]. Two dominant UC processes occur in Ho^3+^ systems, denoted as UC1 (${\;\;}^{5}I_{7}{,}^{5}I_{7}\to^{5}I_{6}{\;\;}^{5}I_{8}$) and UC2 (${\;\;}^{5}I_{7}{,}^{5}I_{7}\to^{5}I_{5}{\;\;}^{5}I_{8}$). However, due to the exceptionally short lifetime of the ${\;\;}^{5}I_{7}$ energy level, the two processes are often indistinguishable in their net effect and can be treated as a single combined upconversion mechanism [29].

### 2.2 Configuration of the F8 cavity

The working of F8 HDFL operating in CW regime can be understood considering dynamics of this particular type of cavity. The F8 fiber laser is a specific type of ring resonator consists of two interconnected loops forming a F8 configuration [18]. One loop is called nonlinear amplifying loop mirror (NALM) or bidirectional loop in which light propagates in both direction i.e. clockwise and counter clockwise [18]. Similarly, the other loop is called unidirectional loop in which the light propagates in clockwise direction only [18]. In the CW regime, the NALM behaves predominantly as a linear optical element due to the relatively low intracavity power. The reflectivity *R* of the NALM can be expressed as [30].

$$R=4\alpha (1-\alpha )\cos^{2}\left(\frac{\Delta \phi }{2}\right)$$ (1)

Where *α* represents the power coupling ratio of coupler (typically 0.5 for a 50:50 configuration) and Δϕ is the phase difference between clockwise and counter clockwise propagating lights. For CW operation, Δϕ≈0 since the nonlinear phase shift $\phi_{NL}$ is negligible [31].

$$\phi_{NL}=\gamma PL\approx 0$$ (2)

Where *γ* is the nonlinearity coefficient, *P* is the optical power, and *L* is the nonlinear fiber length. The F8 fiber laser fulfills this condition when operating in CW regime because the calculated intracavity peak power remains below the threshold to initiate the nonlinear effects, which is 3.1 W for $\gamma =1.6\;\times \;10^{-3}\;{\text{W}}^{-1}{\text{m}}^{-1}$ and L=20m. The gain medium in HDF provides efficient amplification in 2000 nm optical window when excited with pumps at 1950 nm. The small-signal gain *g*_0_ can be described as [32].

$$g_{0}=\sigma_{e}N_{1}-\sigma_{a}N_{0}$$ (3)

Where $\sigma_{e}$ and $\sigma_{a}$ are the emission and absorption cross-sections respectively, while *N*_1_ and *N*_0_ represent the population densities of the upper and lower levels as illustrated in Fig 1b. The steady-state laser operation occurs when the round-trip gain becomes equal to the total cavity losses. Mathematically, this condition may be written as [32].

$$G=\exp(g_{0}L_{HDF})=\frac{1}{R_{OC}R_{NALM}(1-L_{c})}$$ (4)

Where *L*_*HDF*_ is the Holmium fiber length, *R*_*OC*_ is the output coupler reflectivity, *R*_*NALM*_ is the NALM reflectivity from Eq. 1, and *L*_*c*_ represents cavity losses.

The laser’s emission from unidirectional loop is typically enforced through an optical isolator which breaks the reciprocity condition and suppresses the bidirectional lasing. The isolation ratio $\eta_{iso}$ required for stable unidirectional operation can be estimated as [33].

$$\eta_{iso}>\frac{G_{CCW}}{G_{CW}}\approx \exp(-2\alpha_{PC}L_{eff})$$ (5)

Where $\alpha_{PC}$ represents polarization-dependent loss and *L*_*eff*_ is the effective length through the polarization controller (PC). Moreover, *G*_*CW*_ and *G*_*CCW*_ are the round trip gains of two clockwise and counter clockwise beams inside the F8 cavity, respectively where unidirectional operation (e.g., only clockwise or counter clockwise) is achieved via an optical isolator.

In practical implementation, maintaining pure CW operation requires careful balancing of several parameters, such as.

- The pump power must be kept below the mode-locking threshold *P*_*th*_, which can be estimated from the nonlinear phase shift requirement for mode-locking [34].
  $$P_{th}\approx \frac{\pi }{2\gamma L_{eff}}$$ (6)
- The dispersion map of the cavity should be designed to avoid soliton formation [35].
  $$|\beta_{2}|<\frac{T_{0}^{2}}{2\gamma P_{avg}}$$ (7)
  Where $\beta_{2}$ is the group velocity dispersion (GVD) parameter, *T*_0_ is the pulse width, and $P_{avg}$ is the average power.

The birefringence index should be minimized to prevent nonlinear polarization rotation from initiating mode-locking [33].

$$\Delta n<\frac{\lambda }{L_{HDF}}\sqrt{\frac{P_{th}}{P_{avg}}}$$ (8)

Experimental realization of such lasers typically achieve output powers in the range of hundreds of milliwatts to several watts with SEs in the range of 30-40% relative to the pump power. The exact performance characteristics depend on the HDF composition, cavity design, and pump source characteristics. This comprehensive analysis demonstrates that while the F8 configuration is most famous for its mode-locked operation, careful design can make it operate effectively as a CW laser source, particularly in the important 2000 nm wavelength region where HDF provides efficient gain. The mathematical framework presented here allows for quantitative prediction and optimization of the laser’s performance characteristics in CW operation.

## 3 Optimization of the proposed F8 HDFL

**Fig 2:**
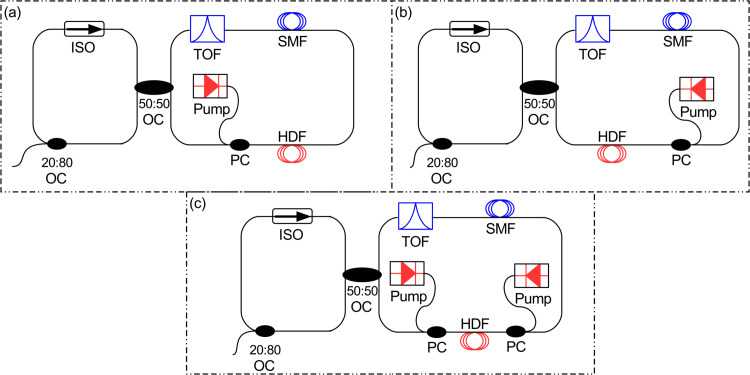
F8 HDFL with different pumping configurations (a) Forward pumping (b) Backward pumping (c) Bidirectional pumping.

Pump configuration, active fiber length, and doping concentration are critical design parameters that directly influence the efficiency, output power, and spectral quality of fiber amplifiers and lasers [36]. An optimized pump configuration ensures effective energy transfer to the gain medium, while the fiber length must be carefully chosen to balance between the gain and losses or nonlinear effects. Similarly, appropriate doping concentrations maximize population inversion without introducing quenching effects, thereby enhancing overall system performance. Fig 2 illustrates various pumping schemes employed to excite the Ho^3+^ ions in the HDF, which serves as the gain medium in the fiber laser system. Forward pumping injects the pump light in the same direction as the intended laser output. Similarly, backward pumping injects the pump light in the opposite direction of the laser output while the bidirectional pumping excites the gain fiber from both ends to create a more uniform population inversion.

**Fig 3:**
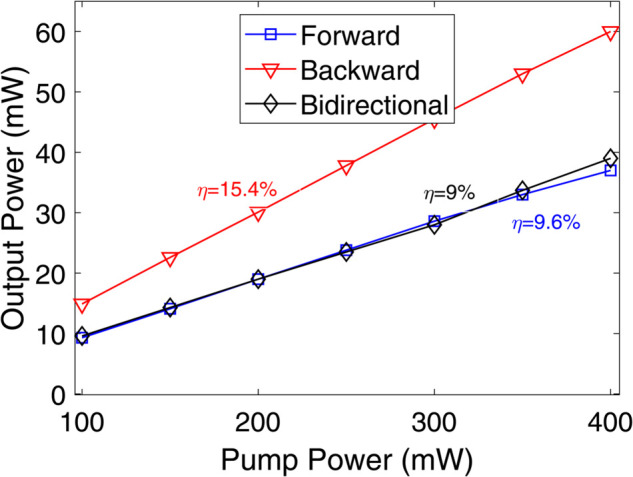
Pump power versus output lasing power plots for various pumping schemes.

First, the pumping scheme is optimized based on the SE values obtained from pump power versus output lasing power characteristics. SE may be defined as the ratio of the increase in laser’s output power to the increase in pump power above the lasing threshold [2]. It represents the fraction of pump power that is absorbed in the gain fiber. Therefore, SE is calculated from the linear region of the output power versus pump power plot and typically expressed in percentage. The pump power is varied from 100 mW to 400 mW for each configuration, and the corresponding output power is measured using an optical power meter (OPM) connected at the output of 20:80 output coupler as illustrated in Fig 2. For this analysis, the HDF length and doping concentration are fixed at 5 m and 40×10^24^ m^−3^, respectively with the tunable output filter (TOF) set at 2030 nm. To ensure a fair comparison, identical parameters are used across all three pumping configurations. Fig 3 presents the pump power versus output lasing power plots obtained for each pumping configuration.

**Fig 4:**
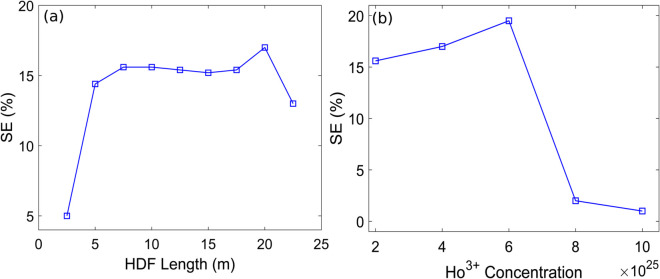
Plots of SE versus (a) HDF length (b) Ho^3+^ doping concentration.

It can be observed that SEs of 9.6%, 15.4%, and 9% are achieved for forward, backward, and bidirectional pumping schemes, respectively. Hence, backward pumping is identified as the optimal configuration for the HDFL, as it delivers the highest SE. Fig 4a displays the plot between the HDF length and SE values with TOF set at 2030 nm while the Ho^3+^ doping concentration is 40×10^24^ m^−3^. It is clearly evident that HDF length of 20 m yields the maximum SE of 17%. Thus, the optimized HDF length for F8 HDFL is 20 m using backward pumping. Similarly, Fig 4b illustrates the plot between the Ho^3+^ doping concentration and SE values with TOF set at 2030 nm while the HDF length is 20 m. It is clearly evident that Ho^3+^ doping concentration of 60×10^24^ m^−3^ yields the maximum SE of 19.5%. Therefore, the optimized Ho^3+^ doping concentration for F8 HDFL is 60×10^24^ m^−3^ using backward pumping scheme. Thus, HDF length of 20 m and doping concentration of 60×10^24^ m^−3^ with backward pumping are determined to be the optimized parameters for the proposed F8 HDFL. Moreover, it is evident from Fig 4 that the SE values exhibit a decreasing trend for longer HDF lengths and higher Ho^3+^ concentrations beyond a certain threshold. The likely causes for this are increased propagation losses in longer HDF lengths and ion-clustering in heavily doped HDF, respectively [29,36].

## 4 Proposed setup

The proposed simulation setup of F8 HDFL employing a single in-band backward pump source has been illustrated in Fig 2b. The proposed F8 HDFL comprises two loops interconnected via 50:50 optical coupler (OC). The right loop functions as a nonlinear amplifying loop mirror (NALM), while the left loop is called unidirectional loop which supports unidirectional clockwise light propagation by employing an optical isolator (ISO). The NALM loop consists of a 20 m long piece of HDF, a 100 m single-mode fiber (SMF) as passive fiber, and an intra-cavity tunable optical filter (TOF) for selecting a specific lasing wavelength within the 2020–2140 nm spectral range. The transfer function (*T*(*f*)) of the filter in terms of transmission (*T*(*f*)) is given as [37].

$$T(f)=10^{\frac{-IL}{20}}H(f)$$ (9)

Here, *IL* is the component’s insertion loss. The return loss (*RL*) of TOF is given by the equation.

$$\text{RL}=10^{\frac{-R_{L}}{20}}$$ (10)

Here, the term *R*_*L*_ is characterized by the parameter return loss of the filter. The HDF acts as the gain medium and is pumped using a 5 W, 1950 nm laser diode coupled with the HDF via a pump combiner (PC). The HDF is the key component in the cavity, with specifications similar to the commercially available model IXF-HDF-PM-8-125, manufactured by iXblue [26,38]. Similarly, the unidirectional loop comprises a polarization-independent isolator (ISO) and a 20:80 output OC. The ISO enforces unidirectional light propagation as discussed earlier while the 20% port of the OC is used to extract the CW laser. An optical power meter (OPM) and optical spectrum analyzer (OSA), connected with 20:80 output OC are used for monitoring the results. The key simulation parameter values utilized in this work are summarized in Table 2.

**Table 2:** Main simulation parameters.

Parameter	Value
Pump wavelength	1950 nm
Pump power	5 W
Core radius of HDF	4 *μ*m
Doping radius of HDF	2 *μ*m
Numerical aperture of HDF	0.3
V-number of HDF	2
Absorption cross-section ($\sigma_{a}$)	0.6×10^−25^ m^2^@2046.7 nm
Emission cross-section ($\sigma_{e}$)	1.8×10^−25^ m^2^@2046.7 nm
Total carrier density (*N*_*t*_)	60×10^24^ m^−3^
Carrier densities at ground and excited states (*N*_*o*_ and *N*_1_)	3×10^24^ m^−3^@50% inversion
Resolution bandwidth	0.01 nm
TOF bandwidth	0.01 nm
Insertion and return loss of TOF	0 and 50 dB
Length of passive SMF	100 m
Effective length (*L*_*eff*_) through PC	5 m
Attenuation	0.2 dB/km
Dispersion	17.6 ps/nm/km
Dispersion slope	0.075 ps/nm^2^/km
Samples per bit	128
Number of samples	256

## 5 Results and discussion

**Fig 5:**
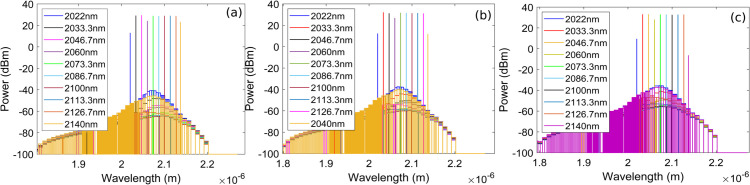
Tuning performance of the F8 HDFL across the 2022–2140 nm wavelength range for various coupling ratios of output coupler (a) 20% (b) 40% (c) 60%. These plots are obtained using optimum parameters as determined in Sect 3.

**Fig 6:**
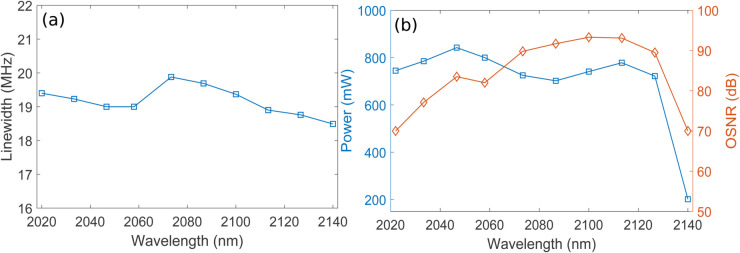
Plots of wavelength versus (a) LWs of different lasing wavelengths for 20% of coupling ratio (b) Average output power and OSNR for 20% of coupling ratio.

**Fig 7:**
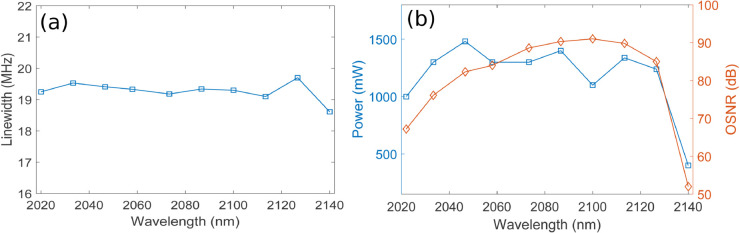
Plots of wavelength versus (a) LWs of different lasing wavelengths for 40% of coupling ratio (b) Average output power and OSNR for 40% of coupling ratio.

**Fig 8:**
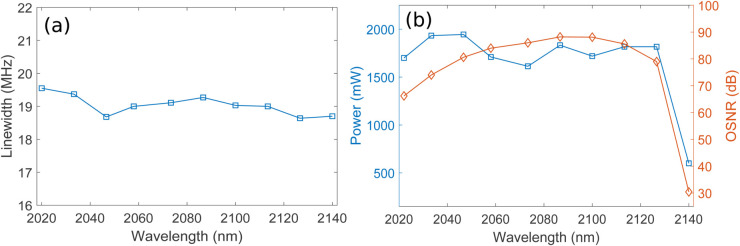
Plots of wavelength versus (a) LWs of different lasing wavelengths for 60% of coupling ratio (b) Average output power and OSNR for 60% of coupling ratio.

The F8 HDFL achieves tuning across the 2022–2140 nm wavelength range, as shown in Fig 5a, Fig 5b, and Fig 5c for coupling ratios of 20%, 40%, and 60% respectively. Tuning of the F8 HDFL is accomplished by sweeping the center wavelength of intracavity TOF in the wavelength range of 2022–2140 nm, which selectively transmits the desired wavelength towards the output coupler while blocking the others. A 1950 nm semiconductor laser diode of 5 W is used in a backward pumping configuration enabling a wide 120 nm tuning span. Minor fluctuations in lasing power across different wavelengths can be observed arising from the variation in Ho^3+^ absorption and emission cross-sections, as illustrated in Fig 1a. Moreover, it is clearly evident from Fig 5a, Fig 5b, and Fig 5c that the lasing power increases with increasing the output coupling ratio. A higher output coupling ratio extracts a larger fraction of the intracavity power, reducing losses, and allowing more of the lasing power to be emitted as output. Fig 6a shows the linewidths (LWs) of different lasing wavelengths corresponding to 2022–2140 nm wavelength range for coupling ratios of 20%. It can be observed that LWs in the range of 18.49-19.7 MHz are obtained corresponding to 2022–2140 nm wavelength range. Similarly, Fig 6b presents the plots of wavelength versus average output power and OSNR corresponding to 2022–2140 nm wavelength range for coupling ratio of 20%. It is clear that highest output power and OSNR of 0.842 W and 93.3 dB are obtained at lasing wavelengths of 2046.7 nm and 2100 nm, respectively. Again, variations in output lasing power and OSNR across the 2022-2140 nm wavelength range can be observed which may be attributed to the wavelength-dependent absorption and emission cross sections of Ho^3+^. It is important to mention here that plots of Fig 6 are obtained using optimized parameters and pump power of 5 W for 20% of coupling ratio. Fig 7a shows the LWs of different lasing wavelengths corresponding to 2022–2140 nm wavelength range for coupling ratios of 40%. It can be observed that LWs in the range of 18.61-19.7 MHz are obtained corresponding to 2022–2140 nm wavelength range. Similarly, Fig 7b presents the plots of wavelength versus average output power and OSNR corresponding to 2022–2140 nm wavelength range for coupling ratio of 40%. It is clear that highest output power and OSNR of 1.5 W and 91 dB are obtained at lasing wavelengths of 2046.7 nm and 2100 nm, respectively. Again, variations in output lasing power and OSNR across the 2022-2140 nm wavelength range can be observed which may be attributed to the wavelength-dependent absorption and emission cross sections of Ho^3+^. It is important to mention here that plots of Fig 7 are obtained using optimized parameters and pump power of 5 W for 40% of coupling ratio. Fig 8a shows the LWs of different lasing wavelengths corresponding to 2022–2140 nm wavelength range for coupling ratios of 60%. It can be observed that LWs in the range of 18.64-19.55 MHz are obtained corresponding to 2022–2140 nm wavelength range. Similarly, Fig 8b presents the plots of wavelength versus average output power and OSNR corresponding to 2022–2140 nm wavelength range for coupling ratio of 60%. It is clear that highest output power and OSNR of 2 W and 88.2 dB are obtained at lasing wavelengths of 2046.7 nm and 2100 nm, respectively. Again, variations in output lasing power and OSNR across the 2022-2140 nm wavelength range can be observed which may be attributed to the wavelength-dependent absorption and emission cross sections of Ho^3+^. It is important to mention here that plots of Fig 8 are obtained using optimized parameters and pump power of 5 W for 60% of coupling ratio. Furthermore, it is also evident from Figs 7b and 8b that average lasing power increases on increasing the coupling ratio which may be attributed to the fact that higher output coupling ratio reduces the intracavity loss, enabling a greater portion of the generated power to be efficiently extracted, thereby increasing the average output lasing power.

**Fig 9:**
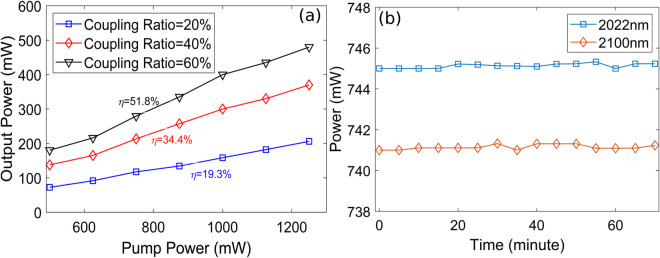
(a) Pump power versus output power plots as a function of coupling ratio (b) Power variation in output lasing power of F8 HDFL for lasing wavelengths of 2022 nm and 2100 nm.

Fig 9a shows pump power versus output lasing power plots as a function of coupling ratio at lasing wavelength of 2046.7 nm considering optimized parameters. SE of 19.3%, 34.4%, and 51.8% are obtained for coupling ratios of 20%, 40%, and 60% respectively. It is evident that SE increases on increasing the coupling ratio of output coupler. The reason behind this trend is that SE increases with a higher output coupling ratio because it reduces the fraction of power lost to internal cavity, such as scattering and absorption, thereby improving the differential efficiency of converting additional pump power into usable output light. Output power stability is an important design artifact of lasers which is critical for applications like material processing, medical procedures, and telecommunications. It minimizes defects, guarantees measurement accuracy, and is a key indicator of the laser’s robustness against environmental disturbances and intrinsic noise. Fig 9b illustrates the average lasing powers at lasing wavelengths of 2022 nm and 2100 nm over fifteen iterations, each recorded at 5-minute intervals using the pump power and coupling ratio of 5 W and 20%, respectively considering optimized parameters. Thus, the time axis corresponds to iteration number × 5 minutes (e.g., first point at 5 minutes, second at 10 minutes, etc.). As shown, a power fluctuation of approximately 0.3 dB is observed for both wavelengths. The power stability of the F8 HDFL is influenced by internal factors such as pump source variations and mode competition, as well as external influences like temperature changes, mechanical vibrations, and ambient environmental conditions. These effects can cumulatively impact the overall power stability over time.

**Fig 10:**
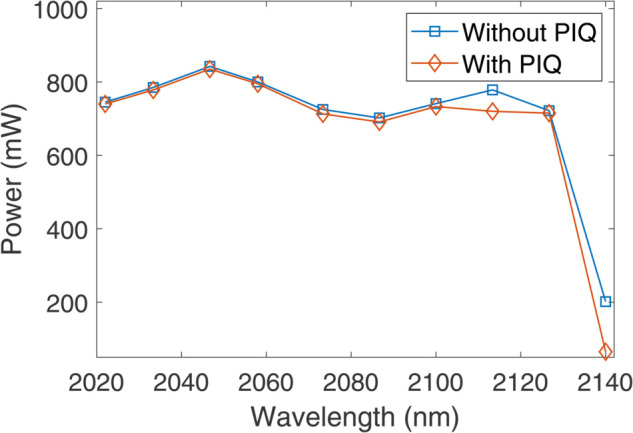
Pump power versus output lasing power plots for various pumping schemes.

Doping the core of an optical fiber with Ho^3+^ ions often results in a non-uniform distribution, where a considerable fraction of the ions are located in close proximity, forming pairs or clusters [29]. In heavily doped HDFs, these ion-pairs can interact and exchange energy in a manner that diminishes the gain build-up in HDF [29]. This energy transfer primarily occurs through two upconversion mechanisms, which are called homogeneous and inhomogeneous upconversion. The latter is also referred to as pair-induced quenching (PIQ) [29]. In homogeneous upconversion, energy transfer occurs between uniformly distributed ions that are spatially well-separated [29]. In contrast, PIQ arises from uneven ion distribution, where energy transfer takes place between closely spaced excited ion-pairs [29]. Both processes contribute to pump power loss and depletion of the excited-state population, thereby reducing the overall gain. To evaluate the effect of PIQ on the performance of the F8 HDFL, we have tuned the F8 HDFL in 2022–2140 nm wavelength range using TOF as did earlier, with and without considering PIQ. We have plotted the wavelength versus output lasing power of the F8 HDFL with and without considering PIQ as illustrated in Fig 10. The analysis is performed using optimized parameters, 5 W of pump power, and 20% of coupling ratio. Table 3 lists two cross-relaxation (CR) coefficients between different energy levels, two homogeneous upconversion (HUC) coefficients between different energy levels, and two ions per cluster for modeling the PIQ in Optisystem. Both CR processes are represented by *K*_2101_ and *K*_1012_ coefficients while both HUC processes are represented by *K*_3101_ and *K*_1013_ coefficients, where 0, 1, 2, and 3 are different energy levels. It can be observed that the inclusion of PIQ leads to a minor reduction in output lasing power. A negligible panelty of around 7 mW is observed in output power of the laser at 2046.7 nm for 20% of coupling ratio. Although the effect of PIQ is negligible at relatively low Ho^3+^ doping concentrations, this analysis confirms that under heavy doping, PIQ can significantly degrade the laser’s performance by reducing the output power.

**Table 3:** Values of various parameters used in the analysis of PIQ.

Parameter	Value
CR coefficient (*K*_2101_)	$2\times 10^{-24}\;m^{-3}s^{-1}$
CR coefficient (*K*_1012_)	$4\times 10^{-23}\;m^{-3}s^{-1}$
HUC coefficient (*K*_3101_)	$7.8\times 10^{-22}\;m^{-3}s^{-1}$
HUC coefficient (*K*_1013_)	$2.3\times 10^{-24}\;m^{-3}s^{-1}$
Ions per cluster	2

## 6 Conclusions

In this work, a watt-level, high optical signal to noise ratio continuous wave Holmium-doped fiber laser operating in the eye-safe 2000 nm window has been designed and analyzed for optical wireless communication applications. The proposed figure-8 cavity-based Holmium-doped fiber laser, incorporating a single in-band backward pump source demonstrates tunability in the 2022–2140 nm wavelength range. Optimization process revealed that HDF length of 20 m and doping concentration of 60×10^24^ m^−3^ with backward pumping were determined to be the optimized parameters. Performance of the laser was evaluated considering the optimized settings. Maximum output powers of 0.842 W, 1.5 W, and 2 W were achieved at 2046.7 nm for coupling ratios of 20%, 40%, and 60%, respectively. The highest slope efficiency of 51.8% was recorded for 60% of coupling ratio at the same wavelength. Optical signal to noise ratio and linewidth values in the range of 30.4–93.3 dB and 18.5–19.9 MHz, respectively confirm the laser’s high spectral quality. A minimal power fluctuation of approximately 0.3 dB over time at selected wavelengths confirms operational stability against environmental disturbances. Additionally, the influence of pair-induced quenching was found to be negligible, causing only a minor output power reduction of around 7 mW at 2046.7 nm. These findings highlight the suitability of the proposed F8 HDFL design for robust and efficient OWC systems.
